# Prognostic value of heart rate variability in atrial fibrillation recurrence following catheter ablation: A systematic review and meta-analysis

**DOI:** 10.3389/fcvm.2022.1048398

**Published:** 2023-02-02

**Authors:** Enyuan Zhang, Shuo Liang, Tianhong Sun, Jing Xu, Fengmin Lu, Dongyan Wu, Jingkun Zhang, Le He, Fan Zhang, Shaobo Fan, Wei Ma

**Affiliations:** ^1^Heart Rhythm Center, Department of Cardiology, Tianjin Chest Hospital, School of Medicine, Nankai University, Tianjin, China; ^2^Department of Radiology, Tianjin Chest Hospital, Tianjin University, Tianjin, China; ^3^Cardiac Function Department, Tianjin Chest Hospital, Tianjin University, Tianjin, China; ^4^Cardiovascular Research Institute, University of California San Francisco, San Francisco, CA, United States

**Keywords:** atrial fibrillation, heart rate variability, recurrence, systematic review, meta-analysis

## Abstract

**Background:**

Atrial fibrillation (AF) has been a worldwide health issue with increasing prevalence and mortality. Recently, increasing attention has been gained to the relationship between heart rate variability (HRV) and the clinical prognosis of AF catheter ablation. We aimed to evaluate the prognostic value of HRV in AF recurrence.

**Methods:**

We systematically searched Web of Science, PubMed, and Embase from inception until 17 August 2022 to conduct the systematic review and meta-analysis. We included the studies reporting the predictive value of HRV parameters for AF recurrence or in which HRV parameters in AF recurrence and non-recurrence groups were individually reported.

**Results:**

Finally, we enrolled 16 studies, including 2,352 patients. Higher rMSSD could independently predict AF recurrence following catheter ablation (OR: 1.02, 95% CI: 1.00–1.04; *p* = 0.03). Higher HF (OR: 1.55, 95% CI: 1.05–2.28; *p* = 0.03) and lower LF/HF (OR: 1.12, 95% CI: 1.03–1.20; *p* = 0.004) could independently predict AF recurrence within 1 year. Higher SDNN (OR: 1.02, 95% CI: 101–1.02; *p* = 0.0006) could independently predict AF recurrence among patients with paroxysmal AF. Almost all HRV parameters within 3 days after catheter ablation and lnHF, lnLF, and rMSSD at 3 months after catheter ablation performed significant differences in AF recurrence and non-recurrence groups.

**Conclusion:**

Heart rate variability, especially higher rMSSD (within short-term and long-term periods), was closely related to recurrent AF following catheter ablation, highlighting the clinical importance of HRV in the prognosis of AF following catheter ablation.

## 1. Introduction

Atrial fibrillation (AF) gradually became the most common arrhythmia with increasing age (1, 2). Patients with AF are at a higher incidence of several major adverse cardiovascular events, including arterial embolism, decompensate heart failure, and even mortality (3). Catheter ablation has been increasingly received as an efficient and even first-line treatment strategy for patients with AF due to its effectiveness at rhythm control when compared with anti-arrhythmic drugs (4, 5). Current catheter ablation has been recommended by several authoritative guidelines for AF management due to the quality of life (QoL) improvement, reduction of heart failure hospitalization, and almost similar prognosis compared with receiving surgical ablation among patients with long-standing persistent AF (6–8). However, some patients with AF still experience AF recurrence after catheter ablation (9).

Heart rate variability (HRV), primarily mediated by cardiac sympathetic and vagal innervation, is a sensitive, quantitative, convincing, and non-invasive index of autonomic neurocardiac function (10, 11). High HRV represents adaptable internal and external stimuli and the dynamic autonomic nervous system (12), while low HRV symbolizes an impaired autonomic nervous ability to maintain homeostasis, which sometimes equals severe cardiac morbidity (13, 14).

However, the relationship between HRV and AF prognosis remains not yet completely determined. There are no meta-analyses supporting a significant relationship between HRV and the increasing incidence rate of AF recurrence following catheter ablation in the general population. Therefore, we performed a meta-analysis to assess the relationship between HRV and AF recurrence rates following catheter ablation.

## 2. Methods

We designed, conducted, and reported the meta-analysis in accordance with guidelines previously introduced (15). All statistical analyses were based on previously published research; therefore, no ethical approval and patient consent are required.

### 2.1. Literature search

We searched the relevant cohort studies from electronic databases including Web of Science, PubMed, and Embase, from inception to 17 August 2022. A combined search term was utilized of “heart rate variability” OR “PNN50” OR “rMSSD” OR “SDNN” OR “low frequency” OR “high frequency” OR “parasympathetic” OR “vagus” OR “sympathetic,” with “atrial fibrillation” and “ablation” OR “pulmonary vein isolation” OR “recurrence.” We only enrolled the studies published in English. As complementation, we further manually searched the references of the related original studies for potentially related articles.

### 2.2. Inclusion and exclusion criteria

We enrolled the studies to fulfill the following inclusion criteria: (1) cohort studies; (2) patients with AF receiving catheter ablation; (3) individually reported HRV parameters in AF recurrence and non-recurrence groups within 3 days or 3 months following catheter ablation procedure as continuous variables OR, and (4) reported the odds ratios (ORs) for AF recurrence as categorical variables.

No restrictions of minimal sample sizes or shortest follow-up duration were applied. The latest research with the longest follow-up duration and the largest sample was enrolled, while different studies reported or shared a part of the same cohort. The most adequately adjusted ORs were extracted when different ORs of HRV were reported.

Exclusion criteria included as follows: (1) animal studies, case reports, reviews, abstracts only, or letters; (2) articles that provide insufficient data on any HRV parameters; and (3) duplicate reports.

### 2.3. Data extracting and quality evaluation

Two authors (EZ and WM) independently performed a literature search, extracted data, and assessed the quality of enrolled studies. The third author (FL) processed the consultation to resolve discrepancies. We finally recorded the following data: first author name, publication year, study region, sample size, study type, ablation procedure method, the proportion of paroxysmal AF, blanking period and follow-up duration after the procedure, age and sex distribution of the included patients, the prevalence rate of hypertension, diabetes mellitus and prior stroke, left ventricular ejection fraction and left atrial diameter of the patients, reported HRV parameters and detailed recorded time point, and AF recurrence rate following catheter ablation. The quality of the included studies was assessed with the Newcastle–Ottawa Scale (NOS) (33). AF recurrence was defined as any episode of AF or atrial tachycardia of at least 30 s recorded with electrocardiogram and 24-h Holter after the blanking period (if mentioned) from ablation.

All the HRV parameters were obtained from the 24-h Holter monitoring. In the time domain, the standard deviation of the normal-to-normal sinus-initiated inter-beat intervals (SDNN), root-mean-square of successive RR-interval differences (RMSSD), and percentage of adjacent NN intervals varying by more than 50 ms (pNN50) were analyzed. In the frequency domain, low frequency (LF), high frequency (HF), and LF/HF ratio were calculated (34).

Low frequency and HF are expressed either by absolute powers, reported in units of ms^2^ (square milliseconds) or natural logarithm (Ln). RMSSD and pNN50 are associated with HF, which represents parasympathetic nerve activity, whereas SDNN is related to LF, which both reflect the total sympathetic and parasympathetic tone activity (35). LF/HF ratio represented the balance of the sympathovagal nerve (Table 1).

**Table 1:** Descriptive characteristics of HRV parameters.

**Acronym (unit)**	**Full name**	**Signification**
**Time-domain**		
SDNN (ms)	Standard deviation normal-to-normal of RR intervals	Correlated with LF power
rMSSD (ms)	Root mean square of successive RR-intervals differences	Associated with HF power and hence parasympathetic activity
pNN50 (%)	Percentage of adjacent NN intervals varying by more than 50 milliseconds	Associated with HF power and hence parasympathetic activity
**Frequency-domain**		
LF (ms2)	Power of the high-frequency band (0.04–0.15 Hz)	Index of both sympathetic and parasympathetic activity, with a predominance of sympathetic
HF (ms2)	Power of the high-frequency band (0.15–0.4 Hz)	Represents the most efferent vagal (parasympathetic) activity to the sinus node
LF/HF	LF/HF ratio	Sympathovagal balance

### 2.4. Statistical analyses

Data of ORs and their corresponding standard errors were calculated according to 95% confidence intervals (CIs) when the value of HRV parameters in predicting AF recurrence was provided. Mean value and standard deviation were performed when HRV parameters of the AF recurrence group and non-recurrence group were offered individually as continuous variables. We performed Cochrane's *Q*-test and *I*^2^ test to evaluate the heterogeneity (36). HRV parameters within 3 days or 3 months following catheter ablation in the AF recurrence group and non-recurrence group were compared with each other. We applied the random-effect model when significant heterogeneity exists among enrolled studies (*I*^2^ > 50% or *p* < 0.05), or we used the fixed-effect model (36). Sensitivity analyses were performed to assess the outcome stability (37). Subgroup analyses were performed to assess the potential influences of type of AF, patient ethnicity, and follow-up duration on results. We performed the funnel plots to show the publication bias. The asymmetrical plot was regarded as the existence of publication bias (38). A *p*-value of < 0.05 was considered a significant statistical difference. We used RevMan (Version 5.3) and RStudio software (Version 1.3.959) for statistical analysis.

## 3. Results

### 3.1. Results of database search and study inclusion

**Figure 1:**
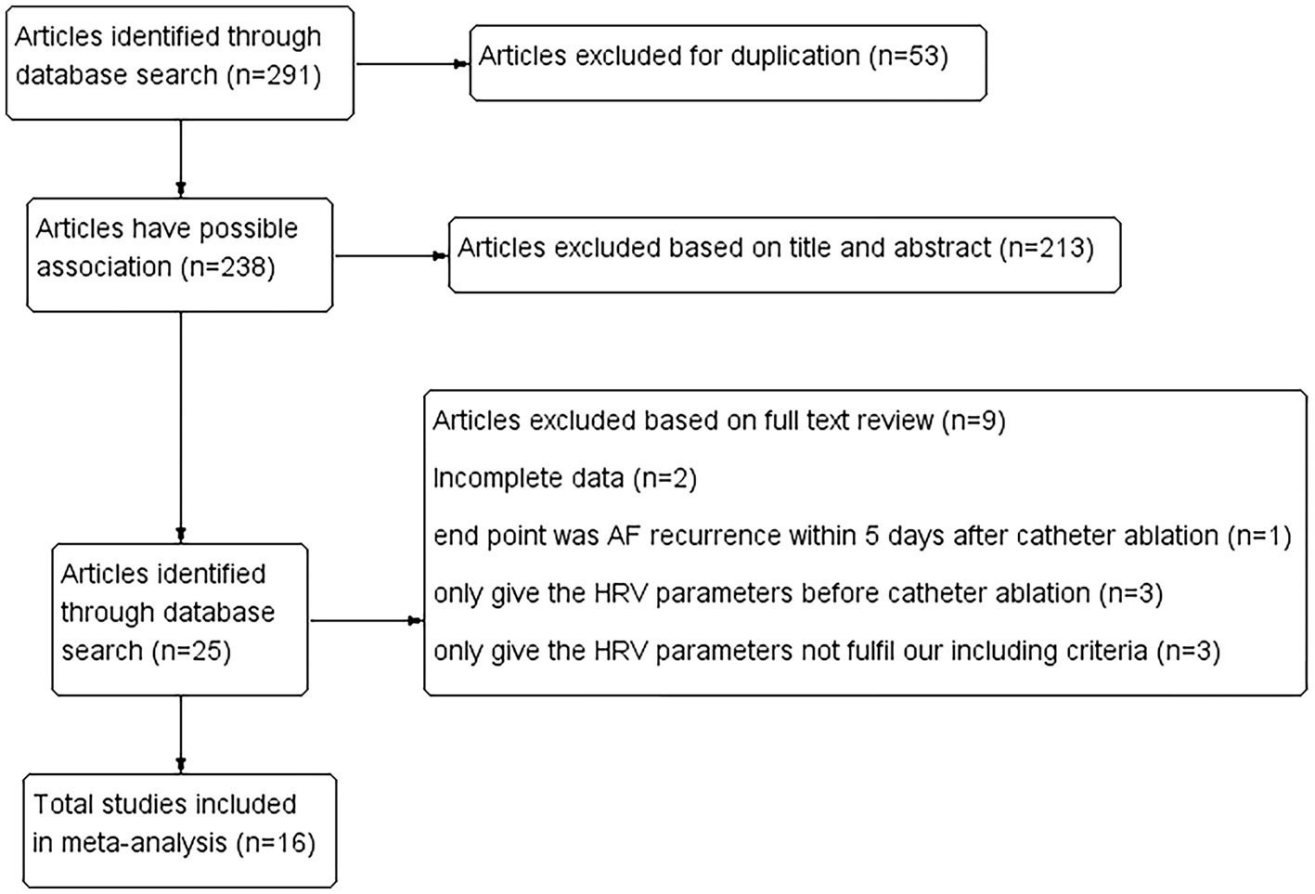
Literature search and identification process.

The literature search and identification process are shown in Figure 1. A total of 238 studies were initially enrolled by excluding duplicated research, and 213 were further eliminated through screening abstracts. Full-text readings were processed among the remaining 25 studies. Of them, nine studies were finally eliminated (Figure 1). Finally, 16 cohort studies from China, Japan, America, Korea, Russia, Serbia, and Canada, including 2,352 patients with AF who received catheter ablation were enrolled (16–26, 28–32).

### 3.2. Study characteristics and quality evaluation

The clinical characteristics, design details, and HRV parameters of the enrolled studies are shown in Table 2. Overall, two (22, 23) of them were prospective studies, while the other three were retrospective cohorts. The sample sizes varied from 42 (16) to 614 (19). Of them, nine studies included paroxysmal AF (20, 22–24, 26, 28, 30–32), while the other two included both paroxysmal and persistent AF. Patients in all included research underwent pulmonary vein isolation [linear, triggers (28, 31), cavotricuspid isthmus (16, 25, 26, 30), mitral isthmus (26), complex fractionated atrial electrogram (21, 24, 30), superior vena cava (19, 26, 28, 31), ganglionated plexi (24) ablation, or BOX (29) if necessary] as the ablation strategy for AF. The follow-up duration varied between 6 (16) and 62 (26) months. The recurrence incidence rate varied between 13.3% (32) and 69.7% (18) (Table 3). The NOS of all enrolled articles ranged from seven to nine (Table 2), prompting good research quality.

**Table 2:** The characteristics of all studies included in the systematic review.

**Study**	**Region**	**Sample size**	**Study type**	**Ablation procedure**	**Paroxysmal AF, %**	**Follow-up (months)**	**NOS**
Hao et al. (16)	China	42	NA	PVI (CTI)	59.5	6	7
Higuchi et al. (17)	America	60	R	PVI (linear)	53.3	12	7
Jian et al. (18)	China	337	R	NA	NA	12	7
Jin et al. (19)	China	614	R	PVI (SVC)	86.3	41	8
Kanda et al. (20)	Japan	56	NA	CRYO (linear)	100	10	7
Kang et al. (21)	Korea	144	NA	PVI (linear CAFE)	83.3	20	8
Liu et al. (22)	China	72	P	PVI (linear)	100	12	8
Marinković et al. (23)	Serbia	100	P	PVI (linear)	100	33	9
Pokushalov et al. (24)	Russia	62	NA	PVI (GP+CAFE)	100	12	7
Seaborn et al. (25)	Canada	83	R	PVI (CTI)	67.5	12	7
Wang et al. (26) SPVI	China	64	NA	PVI (SVC MI CTI)	100	62	8
Wang et al. (27) CPVI		111					
Yamada et al. (28) SPVI	Japan	50	R	PVI (SVC triggers)	100	12	8
Yamada et al. (28) CPVI		51					
Yamaguchi et al. (29)	Japan	92	R	BOX	75.0	16	7
Yanagisawa et al. (30)	Japan	235	R	PVI+CRYO (linear CAFE CTI)	100	15	8
Yoshida et al. (31)	Japan	77	NA	PVI (SVC triggers)	100	12	7
Zhu et al. (32)	China	102	NA	PVI	100	24	8

CFAE, Complex fractionated atrial electrogram; CRYO, Cryoballoon; R, Retrospectively; P, Prospectively; N/A, not available; GP, ganglionated plexi; CTI, cavotricuspid isthmus; SPVI, segmental pulmonary vein isolation; CPVI, circumferential pulmonary vein isolation; SVC, superior vena cava; MI, mitral isthmus; NOS, Newcastle–Ottawa Scale.

**Table 3:** Patient clinical and ultrasonographic characteristics, pooled from studies included in the meta-analysis.

**Study**	**Mean age**	**Female, %**	**Prior stroke,%**	**Diabetes mellitus, %**	**Hyper- tension, %**	**LAD, mm**	**LVEF, %**	**Blanking period (month)**	**Recurrence incidence, %**	**HRV parameters**	**Holter recording time (post-ablation)**
Hao et al. (16)	58	38.1	NA	NA	NA	43.0	57.4	NA	40.5	SDDN	1,3,6 months
Higuchi et al. (17)	65	41.7	10.0	18.3	56.7	NA	NA	3	43.3	SDNN	1 day
Jian et al. (18)	55	46.6	NA	15.7	NA	NA	NA	3	69.7	SDNN; rMSSD; PNN50; LF; HF; LF/HF	2-3 days
Jin et al. (19)	58	26.7	8.0	10.1	44.0	39.9	64.0	3	36.2	SDNN; rMSSD; LF; HF; LF/HF	3,12,24 months
Kanda et al. (20)	63	26.8	1.8	19.6	62.5	36.4	71.2	NA	17.9	rMSSD; LF; HF; LF/HF	3,6 months
Kang et al. (21)	57	29.2	9.7	15.3	49.3	41.0	64.0	3	22.9	rMSSD; HF; LF/HF	3,6,12,18,24 months
Liu et al. (22)	58	37.5	NA	15.2	68.1	35.6	62.2	3	18.1	SDNN; rMSSD; LF; HF; LF/HF	3 days
Marinković et al. (23)	56	39.0	9.0	NA	49.0	39.4	62.2	3	38.0	SDNN; rMSSD; PNN50; LF/HF	1 day 1,3,6,12 months
Pokushalov et al. (24)	57	14.5	NA	6.5	16.1	47.0	57.6	3	29.0	SDNN; rMSSD; LF; HF; LF/HF	Immediately and 3,6,9,12 months
Seaborn et al. (25)	57	24.1	NA	14.5	NA	40.9	NA	NA	32.5	SDNN; rMSSD; PNN50; LF; HF; LF/HF	1 hour, 1 day 1.5,3,6,12 months
Wang et al. (26) SPVI	58	37.5	NA	NA	39.1	36.7	58.5	3	32.8	SDNN; rMSSD	2-3 days 1,3,6,12,36,60 months
Wang et al. (27) CPVI	60	24.3			37.8	36.9	58.3		31.5		
Yamada et al. (28) SPVI	58	24.0	NA	NA	NA	34.4	68.0	NA	44.0	SDNN; rMSSD; LF; HF; LF/HF	Immediately and 1,3,6,12 months
Yamada et al. (28) CPVI	59	21.6				34.8	66.6		21.6		
Yamaguchi et al. (29)	60	19.6	NA	NA	56.5	40.3	64.3	3	17.4	rMSSD; PNN50; SDNN; LF; HF; LF/HF	2 days 3,6,12 months
Yanagisawa et al. (30)	62	29.8	NA	NA	NA	36.6	64.5	3	19.1	SDNN; LF; HF; LF/HF	1,3,6,12 months
Yoshida et al. (31)	59	23.4	NA	NA	NA	34.3	67.3	NA	33.8	SDNN; rMSSD; LF; HF; LF/HF	Immediately and 1,3,6,12 months
Zhu et al. (32)	64	41.2	NA	10.8	NA	39.8	62.5	3	13.3	SDNN; PNN50 rMSSD; LF; HF; LF/HF	1 day

LVEF, left ventricular ejection fraction, LAD, left atrial diameter; AF, atrial fibrillation; N/A, not available; HF, high frequency; LF, low frequency; SDNN, standard deviation of all NN intervals; PNN50, percentage of adjacent NN interval differences >50 ms; rMSSD, root mean square of the differences between adjacent NN intervals.

### 3.3. Association between HRV and AF recurrence after catheter ablation

Notably, nine studies (16, 18, 19, 21, 22, 24, 26, 30, 31) referred to the potential value of different HRV parameters within 3 days after catheter ablation (more than three articles mentioned SDNN, rMSSD, HF, and LF/HF) in predicting AF recurrence following catheter ablation.

**Figure 2:**
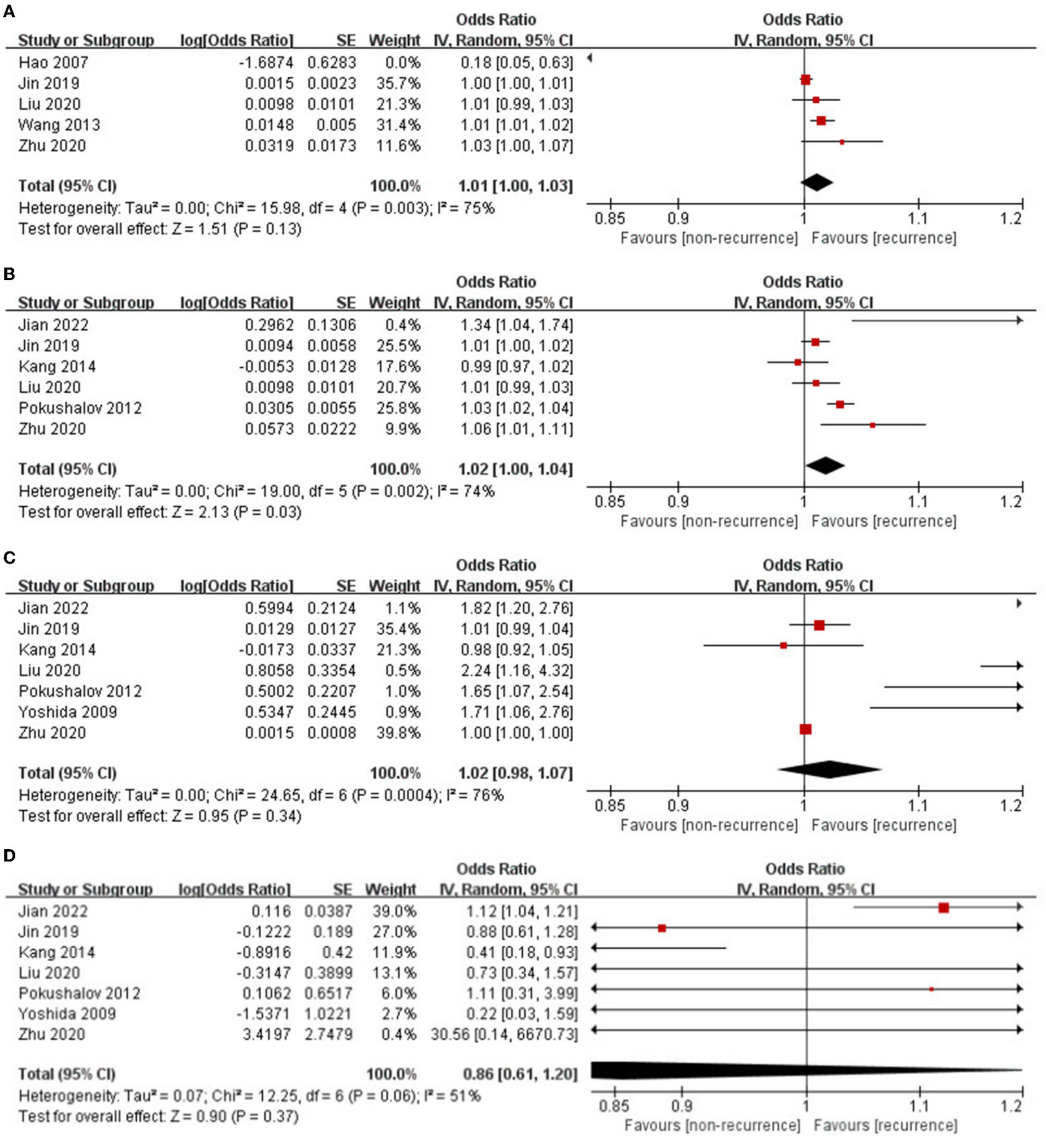
**(A–D)** Association between HRV and AF recurrence after catheter ablation.

The pooled OR for SDNN in predicting AF recurrence after catheter ablation was 1.01 (95% CI: 1.00–1.03; *p* = 0.13, Figure 2A). All five researchers came from China. Significant heterogeneity existed among the enrolled researchers (*I*^2^ = 75%, P for Cochrane's *Q*-test = 0.003). Sensitivity analyses reached similar results. Subgroup analysis enrolled three studies of patients with purely paroxysmal AF, which showed that SDNN could independently predict AF recurrence (OR: 1.02, 95% CI: 1.01–1.02; *p* = 0.0006).

The rMSSD was independently related to an increased incidence rate of AF recurrence following catheter ablation (OR: 1.02, 95% CI: 1.00–1.04; *p* = 0.03, Figure 2B). Significant heterogeneity was observed among the included studies (*I*^2^ = 74%, P for Cochrane's *Q*-test = 0.002). Sensitivity analyses reached similar results.

The pooled OR for HF in predicting AF recurrence following catheter ablation was 1.02 (95% CI: 0.98–1.07; p = 0.34, Figure 2C); further subgroup analysis enrolled four studies of patients with purely paroxysmal AF, which showed that HF still could not independently predict AF recurrence (OR: 1.49, 95% CI: 0.98–2.26; p = 0.06). Significant heterogeneity existed among the enrolled studies (*I*2 = 76%, P for Cochrane's *Q*-test < 0.0001). Sensitivity analyses reached similar results. Subgroup analysis enrolled four studies with a relatively short follow-up period (<12 months) and showed that HF could independently predict AF recurrence (OR: 1.79, 95% CI: 1.41–2.27; *p* < 0.00001).

The pooled OR for LF/HF in predicting AF recurrence following catheter ablation was 0.86 (95% CI: 0.61–1.20; *p* = 0.37, Figure 2D). Medium heterogeneity was observed among the included studies (*I*^2^ = 51%, *P* for Cochrane's *Q*-test = 0.06). Sensitivity analyses omitting one study at a time were found when the study by Jian et al. (18) with the highest recurrence rate (69.7%) was omitted, the heterogeneity sharply decreased (*I*^2^ = 23%, P for Cochrane's *Q*-test = 0.26). Subgroup analysis enrolled four studies with a relatively short follow-up period (12 months) which showed that LF/HF could independently predict AF recurrence (OR: 1.12, 95% CI: 1.03–1.20; *p* = 0.004).

In summary, higher rMSSD could independently predict AF recurrence following catheter ablation. Higher HF and lower LF/HF could independently predict AF recurrence within 1 year. Higher SDNN could independently predict AF recurrence among patients with paroxysmal AF.

### 3.4. Difference in HRV parameters within 3 days after catheter ablation compared with baseline in the AF recurrence group and non-recurrence group

**Figure 3:**
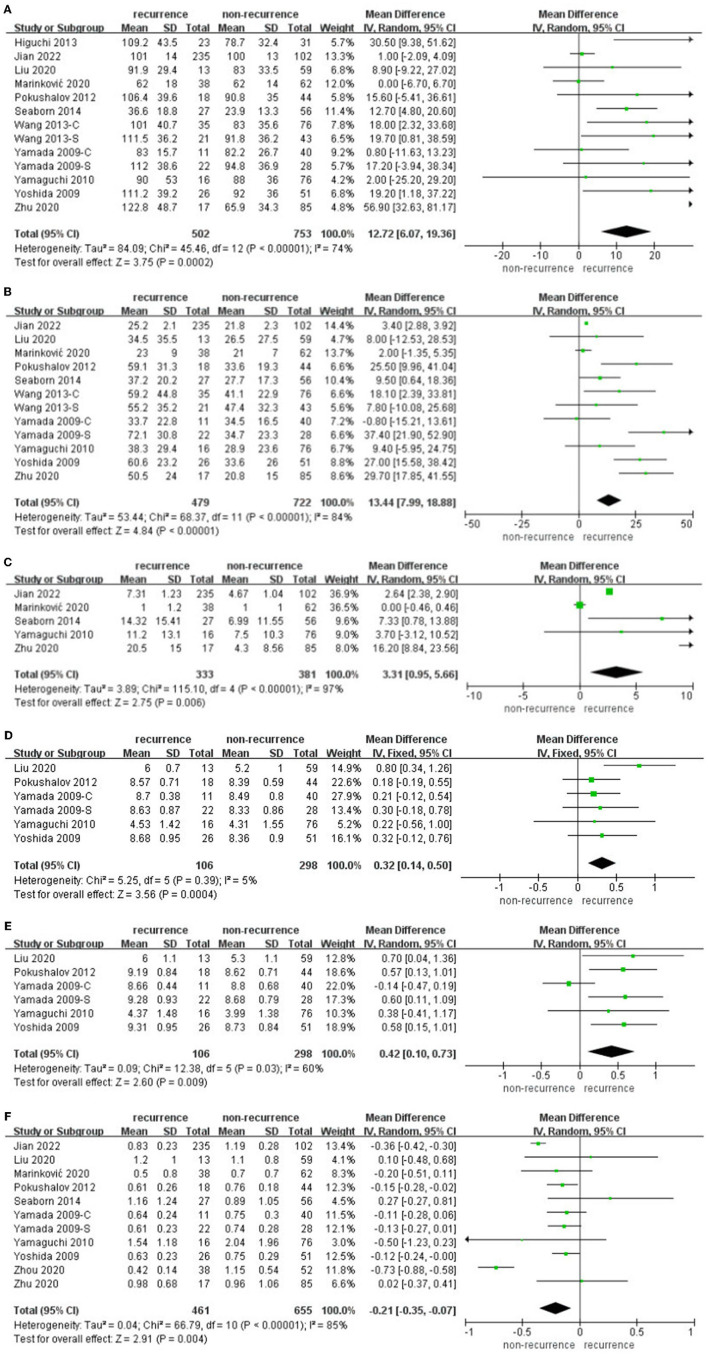
**(A–F)** Difference in HRV within 3 days after catheter ablation compared with baseline in the AF recurrence and non-recurrence group.

Some articles (17, 18, 22–26, 28, 29, 31, 32) reported different HRV parameters within 3 days after catheter ablation in the AF recurrence group and non-recurrence group separately. The data pooled for comparing SDNN difference were 12.72 (95% CI: 6.07–19.36, *p* = 0.0002, *I*^2^ = 74%, Figure 3A), while rMSSD difference was 13.44 (95% CI: 7.99–18.88, *p* < 0.00001, *I*^2^ = 84%, Figure 3B), PNN50 difference was 0.32 (95% CI: 3.31–5.66, *p* = 0.006, *I*^2^ = 97%, Figure 3C), lnLF difference was 0.32 (95% CI: 0.14–0.50, *p* = 0.0004, *I*^2^ = 5%, Figure 3D), lnHF difference was 0.42 (95% CI: 0.1–0.73, *p* = 0.009, *I*^2^ = 60%, Figure 3E), and LF/HF difference was −0.21 (95% CI: −0.35 to −0.07, *p* = 0.004, *I*^2^ = 85%, Figure 3F).

Sensitivity analyses omitting one study at a time in individual meta-analyses all retrieved similar results. Almost all HRV parameters within 3 days after catheter ablation performed significant differences in AF recurrence and non-recurrence groups.

### 3.5. Difference in HRV parameters at 3 months after catheter ablation compared with baseline in the AF recurrence group and non-recurrence group

Some articles (20, 23–25, 29, 30) reported different HRV parameters at 3 months after catheter ablation individually in the AF recurrence group and non-recurrence group separately.

**Figure 4:**
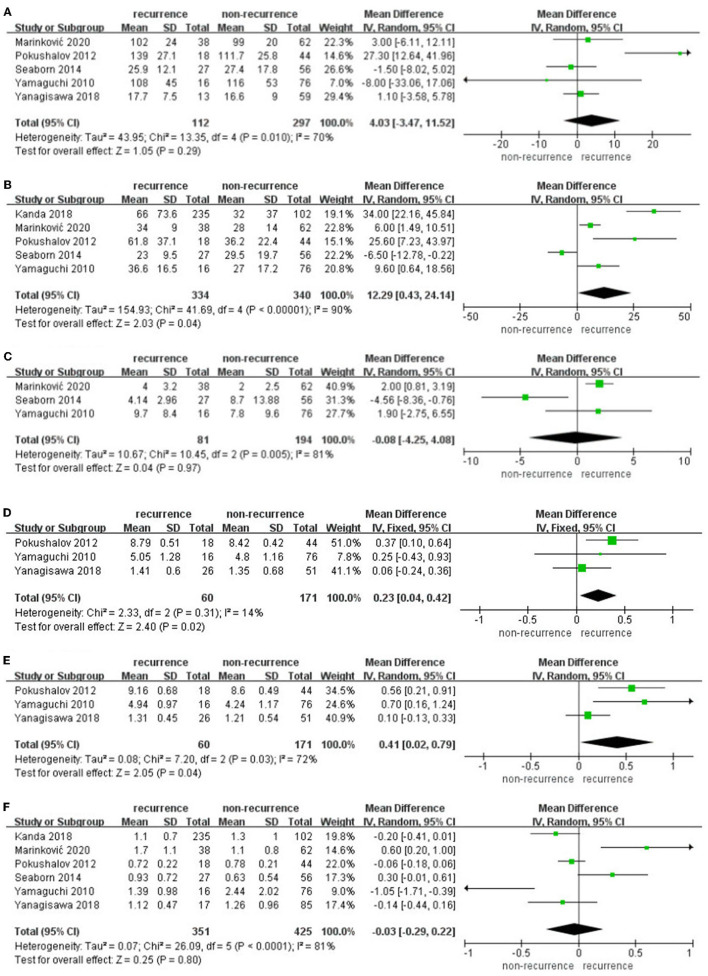
**(A–F)** Difference in HRV parameters at 3 months after catheter ablation compared with baseline in the AF recurrence and non-recurrence group.

The data pooled for comparing SDNN difference at 3 months in patients suffering recurrence and free from recurrence were 4.03 (95% CI: −3.47 to 11.52, *p* = 0.29, *I*^2^ = 70%, Figure 4A). Subgroups according to the ethnicity of the patients, AF type, and follow-up duration did not show any significant influence. rMSSD difference at 3 months in patients suffering recurrence and free from recurrence was 12.29 (95% CI: 0.43–0.42, p = 24.14, *I*^2^ = 90%, Figure 4B). PNN50 difference at 3 months in patients suffering recurrence and free from recurrence was −0.08 (95% CI: −4.25 to 4.08, p = 0.97, *I*^2^ = 81%, Figure 4C); only three articles were enrolled; hence, subgroup analysis was not further performed. lnLF difference at 3 months in patients suffering recurrence and free from recurrence was 0.23 (95% CI: 0.04–0.42, *p* = 0.02, *I*^2^ = 14%, Figure 4D), lnHF difference at 3 months in patients suffering recurrence and free from recurrence was 0.41 (95% CI: 0.02–0.79, *p* = 0.04, *I*^2^ = 72%, Figure 4E), LF/HF difference at 3 months in patients suffering recurrence and free from recurrence was−0.03 (95% CI: −0.29 to 0.22, *p* = 0.80, *I*^2^ = 81%, Figure 4F), and subgroup according to the ethnicity of the patients, AF type, and follow-up duration did not show any significant influence.

Sensitivity analyses omitting one study at a time in individual meta-analyses all retrieved similar results. lnHF, lnLF, and rMSSD at 3 months after catheter ablation performed significant differences in AF recurrence and non-recurrence groups.

### 3.6. Publication bias

Publication bias exploration was conducted through meta-analysis, including more than five studies. Funnel plots of the association between HRV parameters and AF recurrence following catheter ablation were asymmetrical on visual inspection, indicating a high risk of publication bias (figures not shown).

## 4. Discussions

In this meta-analysis, we found that higher rMSSD could independently predict AF recurrence following catheter ablation. Higher HF and lower LF/HF could independently predict AF recurrence within 1 year. Higher HF and SDNN could independently predict AF recurrence among patients with paroxysmal AF. Almost all perioperative HRV parameters performed significant differences in AF recurrence and non-recurrence groups, while only lnHF, lnLF, and rMSSD at 3 months after catheter ablation performed significant differences in AF recurrence and non-recurrence groups. Further sensitivity analysis confirmed the stability of our results. Taken together, these results suggested that HRV, especially for rMSSD, was an independent predictive factor in AF recurrence following catheter ablation. These findings should be further validated in larger-scale prospective studies. To the best of our knowledge, this is the first meta-analysis assessing the association between HRV and AF recurrence following catheter ablation.

Heart rate variability, as a momentous means for quantitative evaluation of autonomic nervous activity nowadays, is concerned with the tiny temporal change of time among adjacent cardiac cycles. The autonomic nervous activity dysfunction could trigger and maintain AF episodes by increasing the left atrial electric heterogeneity. The autonomic nervous system tension change occurs in the onset, development, persistence, and complexity of AF (39, 40). Some patients with AF could not benefit from catheter ablation; the involvement of the autonomic nervous system in the AF propagation may give a satisfactory explanation. Different improvement degrees following AF intervention may help screen patients with autonomic dysfunction and potentially poor prognosis (41). During a mean follow-up of 19.4 years among 11,715 adults in a large cohort, lower SDNN (HR: 1.14, 95% CI: 1.08–1.21), RMSSD (HR: 1.07, 95% CI: 1.01–1.14), HF (HR: 1.12, 95% CI: 1.06–1.17), LF (HR: 1.17, 95% CI: 1.11–1.23) (above HR are expressed per standard deviation lower HRV measures), as well as increased LF/HF (HR: 1.08, 95% CI: 1.03–1.14), were all independently associated with a higher incidence of AF (42). In patients free from AF recurrence, the LnHF decreased and LF/HF increased significantly after box isolation. LnHF was significantly lower in patients who maintained sinus rhythm following surgery (29). HRV appears to show a close relationship with AF recurrence following catheter ablation (18). Lower ΔLF and ΔHF following catheter ablation both predict AF recurrence (22). Reducing overall vagosympathetic activity and minimizing imbalance between the sympathetic/parasympathetic nerve system both contributed to the prevention of AF from recurrence following catheter ablation (20). High parasympathetic tones accurately predict pulmonary vein reconnection among recurrent patients with AF receiving redo catheter ablation, no matter on vein-per-vein analysis or case-per-case analysis (43). The degree and sustained duration of parasympathetic denervation after catheter ablation were both proved related closely to AF recurrence (23). Ln HF 12 months after catheter ablation still predicted AF recurrence (30).

Three decades ago, researchers from Japan demonstrated that the quantitative relationship between the dispersion of refractoriness and the atrial effective refractory period (ERP) could predict increased atrial vulnerability (44). Animal studies found that increased intrinsic vagus tension acts a crucial role in AF triggering by several potential independent mechanisms, a combination of right atrial pacing with 500 beats/min and right cervical vagus trunk simulation in canines resulted in higher AF induction rate and longer AF duration, along with shorter atrial ERP and greater dispersion of ERP, comparison to purely right atrial pacing with 500 beats/min (45). Stimulation of the vagus nerve could increase the frequency and prolong the AF episodes duration *in vivo* by shortening atrial ERP and reducing interatrial conduction velocity (46).

In addition, extrinsic cardiac nerve stimulation from the spinal cord could increase AF inducibility by facilitating the vagus stimulation and attenuating the reaction on left stellate ganglion stimulation (47). Catheter ablation evokes differentiated sympathetic responses directed to the heart and skeletal muscles at different time points. During the procedure, cardiac parasympathetic activity raised and muscle sympathetic nerve activity decreased, while 1 day after the procedure, cardiac parasympathetic activity fell and muscle sympathetic nerve activity raised, which prompted some afferent feedback existing between the cardiac and peripheral autonomic nervous system (48).

A late recent review article found that LF/HF and lower total power could individually be predictors for intraoperative hypotension under spinal and general anesthesia during cardiopulmonary operation (including coronary artery bypass graft, valve surgery, and pulmonary resection) (49).

Pulmonary vein isolation gives rise to remarkable and sustained autonomic nerve alteration reflected by HRV parameters, which were associated closely with procedural outcomes and independent of the ablation method (50). Cardiac ganglionated plexi stimulation by activating the autonomic nervous system induces pulmonary vein triggers and promotes atrial arrhythmogenicity and local reentries at the left atrium–pulmonary vein junction (51). Ablating ganglionated plexi areas around the left atrium extensively, along with pulmonary vein antrum isolation, could enhance the autonomic nerve system denervation through fat pad modification and confers a significantly lower incidence rate of AF recurrence (17, 52). Injection of CaCl2 into the four major atrial ganglionated plexi could significantly reduce the risk of postoperative new-onset atrial fibrillation after cardiac surgery. HF and LF both decreased in the CaCl2 group without the imbalance of the sympathetic/parasympathetic nerve system, which prompts the Ca-mediated neurotoxicity and could inhibit the ganglionated plexi function and arrhythmia onset (27). The recent multicenter randomized clinical trial ERADICATE-AF concluded that a combination of renal denervation and catheter ablation significantly decreased the incidence of AF recurrence within 1 year among patients with paroxysmal AF and hypertension (53).

## 5. Limitations

Several limitations exist in our study. First, this is a meta-analysis enrolling observational cohort studies with heterogeneous design, follow-up duration, ethnicity, and sample sizes. Second, some of the enrolled researchers did not provide the appropriate data in the format required. We failed to obtain available data via conventional data conversion, which may lead to selection bias. Some other selection biases may originate from the exclusion of abstract only. Third, although our meta-analysis enrolled 16 studies, articles referred to each HRV parameter only include a part of them. The funnel plot revealed the publication bias due to the small number of enrolled articles. Fourth, studies from Africa are not found, which may affect the sample representativeness in terms of ethnicity and region.

## 6. Conclusion

Heart rate variability, especially higher rMSSD (within either short-term or long-term periods), was associated with AF recurrence following catheter ablation, indicating that the autonomic activity may be associated with the AF mechanisms, development, and sustainment of AF.

## Author's note

The manuscript, including related data, figures and tables has not been previously published, and that the manuscript is not under consideration elsewhere.

## Data availability statement

The raw data supporting the conclusions of this article will be made available by the authors, without undue reservation.

## Author contributions

EZ, WM, SL, and TS contributed to the acquisition of data, analysis and interpretation of data, and drafting of the article. JX, FL, and DW aided in formal analysis, methodology, and validation. JZ, LH, FZ, and SF aided in revising the article critically for important intellectual content. All the authors read and gave final approval of the version to be submitted.
